# Satellite-based Estimates of Ambient Air Pollution and Global Variations in Childhood Asthma Prevalence

**DOI:** 10.1289/ehp.1104724

**Published:** 2012-05-01

**Authors:** H. Ross Anderson, Barbara K. Butland, Aaron van Donkelaar, Michael Brauer, David P. Strachan, Tadd Clayton, Rita van Dingenen, Marcus Amann, Bert Brunekreef, Aaron Cohen, Frank Dentener, Christopher Lai, Lok N. Lamsal, Randall V. Martin, ISAAC Phase One, Phase Three study groups

**Affiliations:** 1 MRC-HPA Centre for Environment and Health, King’s College London, United Kingdom; 2 MRC-HPA Centre for Environment and Health, St Georges, University of London, United Kingdom; 3 Dalhousie University, Halifax, Nova Scotia, Canada; 4 University of British Columbia, Vancouver, British Columbia, Canada; 5 University of Auckland, Auckland, New Zealand; 6 European Commission, Joint Research Centre, Ispra, Italy; 7 International Institute for Applied Systems Analysis, Laxenburg, Austria; 8 Institute of Risk Assessment Sciences and Julius Center for Health Sciences and Primary Care, Utrecht, the Netherlands; 9 Health Effects Institute, Boston, Massachusetts, USA; 10 The Chinese University of Hong Kong, Hong Kong, People’s Republic of China; 11 Goddard Earth Sciences Technology and Research, Universities Space Research Association, Columbia, Maryland, USA; 12 NASA Goddard Space Flight Center, Greenbelt, Maryland, USA; 13 Harvard-Smithsonian Center for Astrophysics, Cambridge, Massachusetts, USA

**Keywords:** air pollution, asthma prevalence, children, epidemiology, global, nitrogen dioxide, ozone, particulate matter, satellite observations

## Abstract

Background: The effect of ambient air pollution on global variations and trends in asthma prevalence is unclear.

Objectives: Our goal was to investigate community-level associations between asthma prevalence data from the International Study of Asthma and Allergies in Childhood (ISAAC) and satellite-based estimates of particulate matter with aerodynamic diameter < 2.5 µm (PM_2.5_) and nitrogen dioxide (NO_2_), and modelled estimates of ozone.

Methods: We assigned satellite-based estimates of PM_2.5_ and NO_2_ at a spatial resolution of 0.1° × 0.1° and modeled estimates of ozone at a resolution of 1° × 1° to 183 ISAAC centers. We used center-level prevalence of severe asthma as the outcome and multilevel models to adjust for gross national income (GNI) and center- and country-level sex, climate, and population density. We examined associations (adjusting for GNI) between air pollution and asthma prevalence over time in centers with data from ISAAC Phase One (mid-1900s) and Phase Three (2001–2003).

Results: For the 13- to 14-year age group (128 centers in 28 countries), the estimated average within-country change in center-level asthma prevalence per 100 children per 10% increase in center-level PM_2.5_ and NO_2_ was –0.043 [95% confidence interval (CI): –0.139, 0.053] and 0.017 (95% CI: –0.030, 0.064) respectively. For ozone the estimated change in prevalence per parts per billion by volume was –0.116 (95% CI: –0.234, 0.001). Equivalent results for the 6- to 7-year age group (83 centers in 20 countries), though slightly different, were not significantly positive. For the 13- to 14-year age group, change in center-level asthma prevalence over time per 100 children per 10% increase in PM_2.5_ from Phase One to Phase Three was –0.139 (95% CI: –0.347, 0.068). The corresponding association with ozone (per ppbV) was –0.171 (95% CI: –0.275, –0.067).

Conclusion: In contrast to reports from within-community studies of individuals exposed to traffic pollution, we did not find evidence of a positive association between ambient air pollution and asthma prevalence as measured at the community level.

There is substantial evidence from short-term exposure studies that ambient air pollution plays a role in the exacerbation of asthma symptoms [World Health Organization (WHO) 2006]. In contrast, the evidence concerning asthma and long-term exposure to outdoor air pollution is not coherent. Individual-level studies conducted within communities suggest that traffic-related air pollution is associated with both the incidence and the prevalence of asthma (Health Effects Institute 2010), whereas between-community studies (i.e., those that compare communities) do not observe associations between community-average levels of pollution and asthma prevalence (Anderson et al. 2011; WHO 2006; WHO European Centre for Environment and Health 2005).

The International Study of Asthma and Allergies in Childhood (ISAAC) has obtained, using standardized protocols, data on the prevalence of asthma symptoms in > 2 million primary and secondary school–age children from > 200 communities in nearly 100 countries throughout the world (ISAAC 2011). A study of Phase One ISAAC asthma prevalence data from the mid-1990s restricted to cities with > 100,000 population found no evidence of associations between various measures of asthma prevalence and city-level concentrations of particulate matter (with aerodynamic diameter ≤ 10 µm; PM_10_) estimated using an econometric model (Anderson et al. 2010). Recent developments in the application of satellite remote sensing to ground-level air pollution (Hoff and Christopher 2009; Martin 2008) provide important new opportunities for investigating associations between air pollution and health outcomes on a global scale. To our knowledge, satellite-based estimates of particulate matter have been used to investigate associations with respiratory disease on a local scale (in Hong Kong) only (Lai et al. 2010).

In this study we investigated, on a global scale, associations between the community-level prevalence of children’s asthma symptoms and satellite-based estimates of particulate matter with aerodynamic diameter ≤ 2.5 µm (PM_2.5_), nitrogen dioxide (NO_2_), and modeled ozone (O_3_). In contrast with a previous analysis of Phase One ISAAC data (Anderson et al. 2010), the present analysis uses the Phase Three ISAAC data set, which is more recent and includes more centers. In addition, we investigated PM_2.5_ rather than PM_10_, as well as NO_2_ and O_3_. Finally, we examined for the first time associations between trends in air pollution and changes in prevalence within centers between Phases One and Three.

## Methods

*Asthma data.* Detailed ISAAC protocols are available on the ISAAC Website (ISAAC 2011). For the cross-sectional analysis we used previously published 12-month period prevalence estimates for severe asthma symptoms obtained in ISAAC Phase Three (2000–2003) for children 13–14 years of age in 233 centers in 97 countries, and children 6–7 years of age in 144 centers in 61 countries (Lai et al. 2009). Estimates were based on responses to self-completed questionnaires (ages 13–14 years) and parental report questionnaires (ages 6–7 years). The asthma outcome used throughout our analyses was severe wheezing in the preceding 12 months, defined as at least four attacks of wheeze or at least one episode of speech-limiting wheeze, or sleep disturbance due to wheeze at least once a week, during the preceding 12 months (Lai et al. 2009). For the time trend analysis we used published prevalence data for severe asthma symptoms among children 13–14 years of age from 106 ISAAC centers that participated in both Phase One (mid-1990s) and Phase Three (median time between surveys, 7 years) (Pearce et al. 2007). All collaborating centers obtained ethics approval for their study from their local ethics committee or board. Letters describing the survey were sent to parents of all children. Parental completion of the questionnaire for 6- to 7-year-olds implied informed consent. For the 13- to 14-year-olds, passive consent for the child to complete their own questionnaire at school was used by the great majority of centers.

*Pollution data.* For this analysis, we estimated annual ground-level PM_2.5_ concentrations standardized to 50% relative humidity using satellite-based observations by combining aerosol vertical profiles obtained from the global chemical transport model GEOS-Chem with total column aerosol depth obtained from two spectroradiometers (MODIS and MISR) on the satellite Terra, as discussed and validated in detail elsewhere (Brauer et al. 2012; van Donkelaar et al. 2010). The relation between aerosol optical depth and ground-level PM_2.5_ is complex (Paciorek and Liu 2009). However, a comparison of satellite-based PM_2.5_ estimates with ground-level measurements indicated significant agreement for North America (*r* = 0.77; slope = 1.07; *n* = 1,057) and sites from other parts of the world (*r* = 0.83; slope = 0.86; *n* = 244) (Brauer et al. 2012). The 1 SD of uncertainty in satellite-based PM_2.5_ was 25% as inferred through error propagation of uncertainty in satellite remote sensing of aerosol optical depth, in satellite sampling, and in aerosol vertical profile (Holben et al. 1998; Winker et al. 2010). The inferred 25% uncertainty was validated by comparison with *in situ* measurements over North America (van Donkelaar et al. 2010). Concentrations averaged over 2001–2006 were provided at 0.1° × 0.1° geographic grids (Atmospheric Composition Analysis Group 2010b; van Donkelaar et al. 2010). For the time trend analysis we estimated concentrations for 1990 by scaling 2001–2006 estimates using a GEOS-Chem simulation with anthropogenic emissions for 1990.

We estimated annual mean ground-level NO_2_ concentrations, averaged for 2005, by combining GEOS-Chem NO_2_ profiles with tropospheric NO_2_ columns obtained from the Ozone Monitoring Instrument on the satellite Aura (Atmospheric Composition Analysis Group 2010a; Lamsal et al. 2008).

We modeled 3-month running averages of daily 1-hr maximum O_3_ concentrations for the years 1990 and 2005 using the two-way nested TM5 Global Chemical Transport Model (de Meij et al. 2006; Huijnen et al. 2010; Krol et al. 2005) first at a resolution of 1° × 1° at the source regions and then converted to 0.1° × 0.1° grids using mathematical linear interpolation.

*Climate and other covariates.* We obtained daily mean temperature, monthly precipitation, and water vapor pressure data averaged over the period 1991–2000 for 0.5° × 0.5° grids from the International Panel on Climate Change Data Distribution Centre (Mitchell 2004; Mitchell and Jones 2005).

Gross national incomes (GNI) per capita for 2001 (Atlas method) were provided by the World Bank (World Bank 2009) and where missing (five countries), were imputed using data from the Central Intelligence Agency (2003). We obtained population densities for 2005 from the Center for International Earth Science Information Network (2005) and processed them onto the 0.1° × 0.1° pollution grids.

*Assignment of environmental variables to centers.* Our analyses were restricted to ISAAC Phase Three centers with respiratory data for 13- to 14-year-olds and complete pollution data, which were contained (*n* = 177) or almost contained (*n* = 6) within a 1,000-km^2^ square. We used the geographic center of the study population, identified from a map, to obtain a starting 0.1° × 0.1° grid square and the eight surrounding 0.1° × 0.1° grid squares. To confirm that the starting grid square captured the center of population, we compared its population density with that of the eight surrounding 0.1° × 0.1° grid squares. The square with the highest population density was designated the center grid and used for mapping prevalence estimates to climate, altitude, population, and pollution variables.

*Statistical methods.* The Spearman correlation coefficient was used to investigate unadjusted associations between the center-level variables. Our adjusted analysis focused on the regression slopes between asthma prevalence and pollutant variables across centers within country (cross-sectional analysis: estimating the center-level slope) and across time points within centers (trend analysis: estimating the temporal slope). We investigated these associations using multilevel linear regression models (Langford et al. 1998; Leckie 2010) to account for the clustered nature of the data (i.e., centers within countries, and time points within centers within countries) with explanatory variables parameterized as suggested by Begg and Parides (2003) in order to facilitate the separate estimation of effects at different levels of the data hierarchy (e.g., country level, center level).

**Figure 1:**
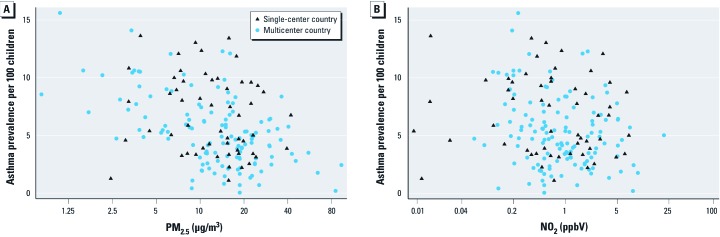
Scatterplots of the association of asthma prevalence at 13–14 years of age with (*A*) PM_2.5_ (µg/m^3^) and (*B*) NO_2_ (ppbV).

Based on histograms (data not shown) both PM_2.5_ and NO_2_ appeared to have positively skewed distributions and were therefore log-transformed before modeling. We used multilevel linear regression rather than multilevel logistic regression because there was evidence of a linear association between asthma prevalence and log PM_2.5_ in both older [Pearson correlation coefficient (*r*) = –0.443; slope = –1.839; *n* = 183; *p* < 0.001] and younger (*r* = –0.341; slope = –1.715; *n* = 85; *p* = 0.001) age groups [Figure 1; see also Supplemental Material, Figure S1 (http://dx.doi.org/10.1289/ehp.1104724)] and because of the problems of overdispersion and scaling associated with the choice of a binomial error structure (Gelman and Hill 2007; Steele 2009).

Country-level variables. Before modeling and for each center-level explanatory variable, we calculated a country-level variable *X̄_i_* defined simply by the formula


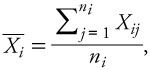
[1]

where *X_ij_* is the value of the explanatory variable for center *j* in country *i* and *n_i_* is the number of centers in country *i* (Begg and Parides 2003).

Cross-sectional analyses. Models for analyses of cross-sectional data included country as a random intercept and fixed effects of the pollutant (log PM_2.5_, log NO_2_, or O_3_), sex (percent boys), the climate variables, and population density in 2005 (including both the center-level and country-level representation of each variable), and GNI per capita in 2001 (available only at country level). These models were used to estimate the absolute change in center-level asthma prevalence (per 100 children) associated with a 10% relative increase in center-level PM_2.5_ or NO_2_ or a 1-ppbV (parts per billion volume) absolute increase in O_3_ (i.e., the center-level regression slope) adjusted for unmeasured effects of country, the fixed country-level effect of the pollutant, and fixed center- and country-level effects of the other explanatory variables.

In addition, for the subset of centers for which there was at least one other center in the same country, we modeled country as both a random intercept and a random slope, thus allowing the estimated center-level effects of pollutants (i.e., center-level regression slopes) to vary among countries. The random intercept and random intercept/random slope models were fitted using XTMIXED in STATA10 (StataCorp, College Station, TX, USA). Significance tests and 95% confidence intervals (CIs) for fixed-effect estimates were based on the standard normal distribution.

For PM_2.5_ and NO_2_, we also added individual cross-level interaction terms to fully adjusted random intercept/random slope models to investigate potential modifying effects of country-level variables on the center-level effects (slopes) of air pollutants. The country-level variables investigated included altitude, latitude, prevalence of current rhinoconjunctivitis, log PM_2.5_, and log NO_2_ (all calculated as in Equation 1) and GNI per capita.

Trend analyses. Models of temporal trend included both center and country as random intercepts, as well as fixed effects of study (i.e., ISAAC phase), the pollutant (log PM_2.5_ or O_3_), and GNI per capita. For Phase One we used GNI per capita for 1992 provided by the World Bank (World Bank 2009), but where these data were missing (five countries) Phase One GNI was set equal to Phase Three GNI. NO_2_ was not available for the trend analysis. The pollutant was represented in models by three variables: *X_ijk_*, the value of the explanatory variable for study *k* in center *j* in country *i*; *X̄_ij_*, the mean value of *X* across studies in center *j* and country *i* (as defined in Equation 2); and *X̄_i_*, the mean value of *X* across centers and studies in country *i* (as in Equation 3, where *n_i_* is the number of centers in country *i*). GNI was represented by two variables: *G_ik_*, GNI per capita for study *k* in country *i*, and *Ḡ_i_*, the mean value of GNI across studies in country *i* (as in Equation 4). This facilitated the separation of temporal effects from center-level and country-level effects. Small numbers precluded any meaningful trend analysis for the 6- to 7-year age group.


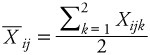
[2]


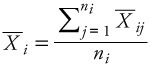
[3]


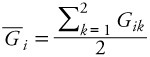
[4]

## Results

*Cross-sectional analysis, ages 13–14 years.* For the cross-sectional analysis of 13- to 14-year-olds, 183 of the 233 centers in 83 of the 97 countries satisfied our inclusion criteria [see Supplemental Material, Figure S2 (http://dx.doi.org/10.1289/ehp.1104724)]. For this population, all three pollutants were positively correlated with population density and negatively correlated with water vapor pressure and rainfall (Table 1). NO_2_ was positively correlated with GNI per capita. Asthma prevalence was negatively correlated with all three pollutants (Table 1, Figure 1) and positively correlated with the three climate variables. The three pollutants were correlated positively with one another, the strongest being PM_2.5_ with O_3_ and the weakest being NO_2_ with O_3_. The Spearman correlation coefficient (*r_S_*) between the one standard deviation of uncertainty in the satellite-based estimate of PM_2.5_ (expressed as percent) and asthma prevalence was *r_S_* = 0.084 (*p* = 0.260).

**Table 1:** Descriptive statistics for the main analytic data set ages 13–14 years (*n* = 183 centers).^a^

Median (interquartile range)	Spearman correlation with						
	Variable	Time period	Asthma prevalence	PM2.5	NO2	O3
Sex (ISAAC Phase Three, ages 13–14 years)												
% boys in sample		≈ 2000–2003		49.2 (47.1–51.5)		–0.082		0.134		0.296#		0.201**
Disease (ISAAC Phase Three, ages 13–14 years)												
Asthma prevalence (%)		≈ 2000–2003		5.05 (3.34–8.04)		—		–0.412#		–0.198**		–0.489#
Climate/altitude												
Daily temperature (°C)		1991–2000		18.7 (12.7–24.9)		0.159*		–0.033		–0.358#		–0.060
Water vapor pressure (hPa)		1991–2000		14.5 (10.8–22.5)		0.209**		–0.183*		–0.372#		–0.196**
Precipitation (mm/month)		1991–2000		81.5 (50.3 –125.1)		0.217**		–0.347#		–0.248#		–0.355#
Altitude (m)		NA		85 (22–458)		0.006		0.099		–0.019		0.102
Economic/population												
GNI per capita (US$)		2001		1,960 (1,020–9,800)		–0.023a		–0.022a		0.537a,#		–0.132a
Population density (thousands per 0.1° × 0.1° grid square)		2005		167 (47.0–514)		–0.038		0.302#		0.408#		0.218**
Pollution												
PM2.5 (µg/m3)		2001–2006		14.6 (8.2–19.4)		—		—		0.468#		0.617#
NO2 (ppbV)		2005		0.77 (0.36–2.00)		—		—		—		0.332#
O3 (ppbV)		2005		53.2 (40.4–61.7)		—		—		—		—
NA, not applicable. aCorrelations for GNI per capita (available only at country level) are with country-level variables (defined as in Equation 1). There are 83 countries. All other correlations are across all 183 centers. *p < 0.05, **p < 0.01, #p < 0.001.												

Cross-sectional analysis of PM_2.5_. The fully adjusted random intercept model estimate (model 3, Table 2)—the estimated change in center-level asthma prevalence associated with a 10% relative increase in center-level PM_2.5_—was small and nonsignificant (–0.016 per 100 children; 95% CI: –0.095, 0.063). The estimated change in center-level asthma prevalence associated with a 10% relative increase in country-level PM_2.5_—the country-level effect—indicates that the association between asthma and PM_2.5_ may differ at different levels of geographical aggregation, and highlights the importance of fitting models that allow for this difference. In this case, the additional estimated effect of country-level PM_2.5_ (defined as in Equation 1) on center-level prevalence was estimated as –0.172 (95% CI: –0.306, –0.038) (Table 2).

**Table 2:** The association of asthma prevalence ages 13–14 years with PM_2.5_ and NO_2_.

Estimated change in center-level asthma prevalence (95% CI) per 100 children per 10% increase						
PM_2.5_			NO_2_		
No.	Model type	Adjustment	Country-level^a^	Center-level^b^	Country-level^a^	Center-level^b^
Using data from 183 centers in 83 countries											
1	Random intercept		Unadjusted		–0.128 (–0.248, –0.009)*		–0.032 (–0.101, 0.037)		–0.032 (–0.092, 0.027)		–0.005 (–0.040, 0.029)
2	Random intercept		Sex, climate, GNI		–0.160 (–0.282, –0.037)*		–0.028 (–0.100, 0.043)		–0.062 (–0.133, 0.009)		–0.002 (–0.037, 0.032)
3	Random intercept		Sex, climate, GNI, population density		–0.172 (–0.306, –0.038)*		–0.016 (–0.095, 0.063)		–0.068 (–0.149, 0.013)		0.012 (–0.031, 0.055)
Restricted to two or more centers per country (128 centers in 28 countries)											
4	Random intercept		Sex, climate, GNI, population density		–0.293 (–0.445, –0.140)#		–0.016 (–0.095, 0.063)		–0.253 (–0.391, –0.114)#		0.012 (–0.031, 0.055)
5	Random intercept/random slopec		Sex, climate, GNI, population density		–0.232 (–0.359, –0.105)#		–0.043 (–0.139, 0.053)		–0.262 (–0.391, –0.133)#		0.017 (–0.030, 0.064)
6	Random intercept/random slope		Sex, climate, GNI, population density, O3		–0.068 (–0.193, 0.058)		–0.020 (–0.132, 0.092)		–0.142 (–0.265, –0.019)*		0.022 (–0.025, 0.069)
7	Random intercept/random slope		Sex, climate, GNI, population density, log(PM2.5) or log(NO2) as appropriate		–0.116 (–0.264, 0.032)		–0.026 (–0.133, 0.081)		–0.163 (–0.309, –0.018)*		0.020 (–0.032, 0.072)
8	Random intercept/random slope		Sex, climate, GNI, population density, O3, log(PM2.5) or log(NO2) as appropriate		–0.004 (–0.139, 0.131)		–0.008 (–0.121, 0.105)		–0.130 (–0.262, 0.002)		0.027 (–0.025, 0.079)
Sex, climate (i.e., temperature, precipitation, water vapor pressure), population density, and pollutants, if included in models were included both as country level (defined as in Equation 1) and center-level variables. GNI per capita was only available at country level. aCountry-level effect: estimate of the association between center-level asthma prevalence and country-level pollutant (defined as in Equation 1). bCenter-level effect: estimate of the within country association between center-level asthma prevalence and center-level pollutant. cTest (likelihood ratio test) for a random slope in PM2.5 (model 5), χ2 = 10.76 (degrees of freedom = 2), p < 0.01; test for a random slope in NO2 (model 5), χ2 = 6.64 (degrees of freedom = 2), p < 0.05. *p < 0.05, #p < 0.001.											

Whereas all 183 centers contributed to the estimation of the country-level effect, only data from those countries with at least two centers could contribute to the estimation of the center-level effect. When the data set was restricted to the 28 countries with at least two centers (*n* = 128) the model fit was improved significantly by allowing the center-level regression slope to vary between countries (model 5), although this had little effect on the overall center-level effect estimate (now the estimated center-level slope for the average country), which was still small and nonsignificant (–0.043; 95% CI: –0.139, 0.053). We found no evidence of any modifying effect on the center-level slope by GNI per capita (*p* = 0.440) or country-level altitude (*p* = 0.664), latitude (*p* = 0.971), prevalence of current rhinoconjunctivitis (*p* = 0.224), log PM_2.5_ (*p* = 0.489), or log NO_2_ (*p* = 0.280).

**Figure 2:**
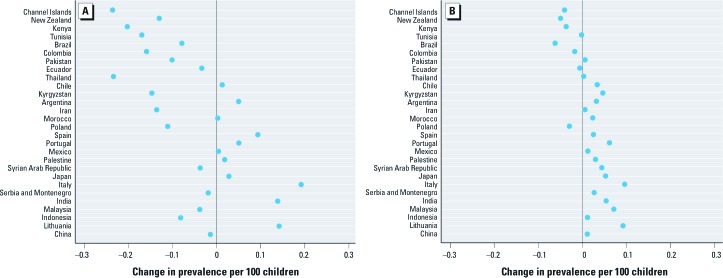
Country-specific estimates of change in center-level prevalence per 100 children 13–14 years of age per 10% increase in center-level PM_2.5_(*A*) and per 10% increase in center-level NO_2_ (*B*). Estimates were obtained from model 5 (Table 2) and sorted by country-level asthma prevalence (defined as in Equation 1) from low (China) to high (Channel Islands).

Figure 2A displays country-specific center-level effect estimates for PM_2.5_ based on model 5 (Table 2). The estimates are sorted by country-level asthma prevalence (defined as in Equation 1) from low (China) to high (Channel Islands) and suggest an inverse association between the gradient of the center-level slope and country-level prevalence.

When we expanded model 5 to include adjustment for NO_2_ and O_3_ (Table 2, models 6–8) the overall center-level effect estimate for PM_2.5_ moved further toward the null.

Cross-sectional analysis of NO_2_. The fully adjusted estimated change in center-level asthma prevalence per 10% increase in center-level NO_2_ (Table 2, models 3 and 4) was small and nonsignificant (0.012; 95% CI: –0.031, 0.055). When we allowed the center-level slope to vary between countries (model 5), the model fit improved significantly although the estimated center-level effect for the average country was still small and nonsignificant (0.017; 95% CI: –0.030, 0.064). Figure 2B shows some suggestion of an inverse association between the gradient of the center-level slope and country-level asthma prevalence. However, we found no evidence of any modifying effects on the center-level slope of other country-level variables including GNI per capita (*p* = 0.944), altitude (*p* = 0.751), latitude (*p* = 0.302), prevalence of rhinoconjunctivitis (*p* = 0.541), log PM_2.5_ (*p* = 0.199), or log NO_2_ (*p* = 0.563).

When we expanded model 5 to include adjustment for PM_2.5_ and O_3_ (Table 2, models 6–8), the center-level effect estimate was little changed.

Cross-sectional analysis of O_3_. Using the random intercept/random slope model with full adjustment (model 5) we estimated that for the average country the change in center-level prevalence (per 100 children) associated with a 1-ppbV increase in center-level ozone was –0.116 (95% CI: –0.234, 0.001) (data not shown).

*Cross-sectional analysis, ages 6–7 years.* The results for children 6–7 years of age are shown in Supplemental Material, Tables S1 and S2, Figure S1 (http://dx.doi.org/10.1289/ehp.1104724). In contrast to our findings for those 13–14 years of age, asthma prevalence among younger children was negatively associated with the percentage of boys in the sample.

In the random intercept model with full adjustment, the estimated change in center-level asthma prevalence per 100 children per 10% increase in center-level PM_2.5_ and NO_2_ was 0.026 (95% CI: –0.116, 0.168) and 0.004 (95% CI: –0.059, 0.067) respectively—both positive and nonsignificant. For O_3_ the estimated change in prevalence per ppbV was –0.128 (95% CI: –0.247, –0.009)—negative and statistically significant. There was no evidence that associations with pollutants in this age group differed among countries (i.e., no significant improvement in the fit of models from allowing center-level slopes to vary between countries).

**Figure 3:**
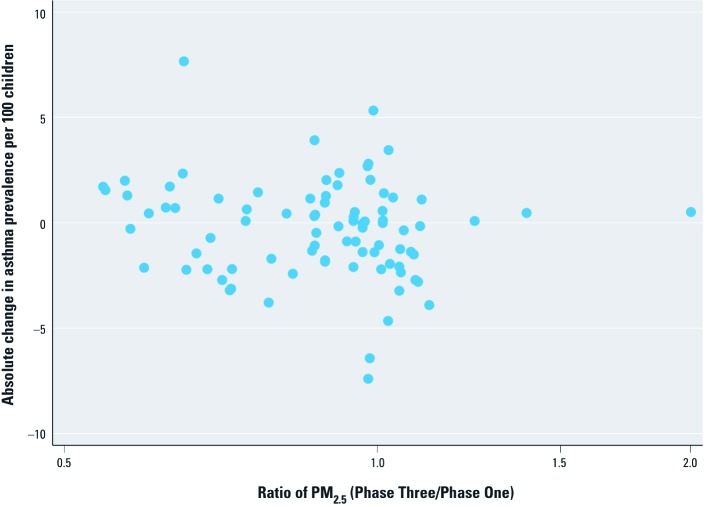
Scatterplot illustrating the association between absolute change (Phase Three – Phase One) in asthma prevalence per 100 children 13–14 years of age and relative change (Phase Three/Phase One) in PM_2.5_.

*Trend analysis.* Eighty-five centers were eligible for the trends analysis of 13- to 14-year-olds [see Supplemental Material, Figure S2 (http://dx.doi.org/10.1289/ehp.1104724)] but these are not representative of the whole sample (see Table 3, notes). Pollution data for the early period were available only for PM_2.5_ and O_3_. A scatterplot (Figure 3) of absolute change in asthma prevalence between phases versus the ratio of PM_2.5_ (Phase Three/Phase One) suggests a weak nonsignificant negative association (Spearman correlation coefficient: *r_S_* = –0.182, *p* = 0.095). Using a random intercept model we estimated the absolute change in asthma prevalence associated with a 10% relative increase in PM_2.5_ over time within center, having adjusted for center (including any effects of center-average PM_2.5_, defined as in Equation 2) and change in GNI per capita. The adjusted estimate was negative but nonsignificant (–0.139; 95% CI: –0.347, 0.068) (Table 3). Using the same approach, the estimated change in asthma prevalence associated with a 1-ppbV increase in O_3_ between phases, adjusted for center (including center-average O_3_, defined as in Equation 2) and change in GNI per capita, was negative and statistically significant (–0.171; 95% CI: –0.275, –0.067). When we attempted to allow these temporal associations to vary between centers using random intercept/random slope models, the model for PM_2.5_ failed to converge, and for O_3_ there was no evidence of any improvement in fit (*p* > 0.05).

**Table 3:** The association between pollutants and asthma prevalence ages 13–14 years: trend analysis based on 85 centers in 50 countries.

Pollutant (increment)	Estimated change in center-level asthma prevalence (95% CI) per 100 children per increment in pollutant				
	Model	Adjustment	Country level^a^	Center level^b^	Center level over time (Phase Three – Phase One)^c^
Random intercept		PM2.5 (10%)		Unadjusted		–0.184 (–0.369, 0.001)		0.155 (–0.092, 0.402)		–0.145 (–0.351, 0.060)
				GNI per capita		–0.200 (–0.379, –0.022)*		0.149 (–0.100, 0.398)		–0.139 (–0.347, 0.068)
Random intercept		O3 (1 ppbV)		GNI per capita		0.201 (0.051, 0.351)**		–0.092 (–0.259, 0.075)		–0.171 (–0.275, –0.067)**
Each pollutant when included in models was included as 3 variables, Xijk, the value of the explanatory variable for study k in center j in country i; X—ij (as defined in Equation 2); and X—i (as defined in Equation 3). GNI per capita, which was available only at country-level was included as two variables, Gik (GNI for study k in country i) and G—i (as defined in Equation 4). A cross-sectional Phase Three analysis for PM2.5 restricted to the 85 centers with both Phase One and Phase Three data and based on a random intercept model with full adjustment, yielded a center-level estimate of 0.21 (0.072, 0.348)** and a country-level estimate of –0.461 (–0.645, –0.277).# aEstimate of the association between center-level asthma prevalence and country-average pollutant (defined as in Equation 3). bEstimate of the within-country association between center-level asthma prevalence and center-average pollutant (defined as in Equation 2). cEstimate of the within-center association between change in center-level asthma prevalence over time (Phase Three – Phase One) and change in center-level pollutant over time (Phase Three – Phase One). *p < 0.05, **p < 0.01.										

## Discussion

The central aim of this study was to investigate the potential role of community-average levels of PM_2.5_, NO_2_, and O_3_ in explaining worldwide variations in childhood asthma prevalence. Neither the cross-sectional nor longitudinal analyses provided any support for a positive association with any of these measures of ambient air pollution. This conclusion is robust to the statistical modeling and control at country level for GNI and at country level and center level for sex, climate, and population density.

Being based on ecological data, our analyses were not designed to investigate associations between air pollution and asthma prevalence at an individual level within centers. An individual-level analysis of ISAAC data found a convincing association between asthma prevalence and proximity to truck traffic (Brunekreef et al. 2009). The disparity between within-community associations at the individual level and between-community associations at the ecological level thus mirrors disparities between individual- and community-level data in the wider literature (Anderson et al. 2011; Health Effects Institute 2010; WHO 2006; WHO European Centre for Environment and Health 2005). In further analyses we plan to investigate this apparent paradox by extending our multilevel models to include individual-level data on proximity to truck traffic, secondhand smoke, and cooking fuels, which are available for a subset of ISAAC Centres.

As part of our current analyses, we estimated country-level effects of pollution, some of which were negative and statistically significant (Table 2). We do not interpret these associations as causally related to ambient air pollution but as representing important clues in the investigation of causes of variations in asthma prevalence between countries. These might be elucidated by identifying potential causal factors that are negatively associated with air pollution such as lifestyle and economic development.

The health hazard of ambient NO_2_ is debated, with most authorities tending to regard it as an indicator of more toxic components of the pollution mixture rather than as toxic per se (WHO 2006). The value of including NO_2_ in our analyses was primarily as a more precise marker of combustion-related pollution than PM_2.5_, which may include noncombustion sources such as dust (Veefkind et al. 2011). We found, however, that NO_2_ was strongly and positively correlated with PM_2.5_. And like PM_2.5_, the within-country center-level association of NO_2_ with asthma symptoms, though more precisely estimated than the estimate for PM_2.5_, was weak and nonsignificant; furthermore, its inclusion in the PM_2.5_ model did not materially influence the center-level estimates for PM_2.5_.

O_3_ is a secondary pollutant which is toxic to the respiratory system at ambient or near ambient concentrations (WHO 2006). It is plausibly linked to asthma prevalence through effects on severity of exacerbations and longer-term airways damage. Our O_3_ concentrations were estimated by the global chemical transport model TM5 but with less confidence than our estimates for PM_2.5_ because the spatial resolution was lower and allowance for the urban titration effect could not be made. The unadjusted correlation for O_3_ across all centers was strongly positive with PM_2.5_ and moderately strongly negative with asthma prevalence; in the main analysis however, the influence of O_3_ on the center-level estimates for PM_2.5_ was small. When it was considered as an explanatory variable, there was some evidence that O_3_ was negatively correlated with childhood asthma both at the center-level (within countries) and over time (within centers). This result is consistent with recent reviews of multicommunity studies of O_3_ and asthma (Anderson et al. 2011).

Our methods for measuring asthma in large populations were limited to questionnaires that attempt to summarize symptoms experienced over a prior period, in this case 12 months. The ISAAC questionnaire has been validated in terms of physician assessment (Jenkins et al. 1996), comparison between surveys of 6- to 7-year-olds and 13- to 14-year-olds carried out independently in the same center, comparisons with independent adult asthma surveys in the same country (Pearce et al. 2000), and comparisons with national hospital admission and mortality rates (Anderson et al. 2008). However, we cannot be certain that the results of this questionnaire would be sufficiently sensitive to reflect any marginal effect of air pollution on asthma exacerbations.

The importance of the trend analysis is that, being within center and by using the same methods, it controls for unknown sources of bias and for unknown or unmeasured confounding factors that do not vary substantially over time. Nevertheless it is even more prone than our cross-sectional analyses to the adverse effects of measurement error. Nonetheless, the nonsignificant negative associations with changes in PM_2.5_ over time are consistent with the nonsignificant negative association with PM_2.5_ obtained in the cross-sectional analysis.

## Conclusion

In this ecological study we did not find evidence of positive community-level associations between the prevalence of asthma and satellite-based estimates of PM_2.5_ and NO_2_ and modeled estimates of O_3_, either cross-sectionally or over time. It is possible that an underlying positive community-level association may have been obscured by insufficient precision in our measures of exposure and outcome and by limited statistical power. Nonetheless, our findings do not support an association between ambient air pollution and asthma prevalence at the community level. The disparity between these findings and those of within-community studies of individuals exposed to traffic pollution remains to be explained.

## Supplemental Material

(1.1 MB) PDF
