# Supplementary material for: Satellite-based Estimates of Ambient Air Pollution and Global Variations in Childhood Asthma Prevalence
Source: Environ Health Perspect. 2012 May 1;120(9):1333–9. doi: 10.1289/ehp.1104724 (PMC3440118; doi:10.1289/ehp.1104724)
Supplement: (1.1 MB) PDF [file ehp.1104724.s001.pdf]

Supplemental Material

Satellite-based Estimates of Ambient Air Pollution and Global Variations in Childhood Asthma Prevalence

H Ross Anderson[1], Barbara Butland[2], Aaron van Donkelaar[3], Michael Brauer[4], David P Strachan[2], Tadd Clayton[5], Rita van Dingenen[6], Marcus Amann[7], Bert Brunekreef[8], Aaron Cohen[9], Frank Dentener[6], Christopher Lai[10], Lok N Lamsal[3,11,12], Randall Martin[3,13], and the ISAAC Phase One and Phase Three study groups.

[1] MRC Centre for Environment and Health, King's College London, UK

[2] MRC Centre for Environment and Health, St Georges, University of London, UK

[3] Dalhousie University, Halifax, NS, Canada

[4] University of British Columbia, Vancouver, BC, Canada

[5] University of Auckland, Auckland, New Zealand

[6] European Commission, Joint Research Centre, Ispra, Italy

[7] International Institute for Applied Systems Analysis, Austria

[8] Institute of Risk Assessment Sciences and Julius Center for Health Sciences and Primary Care, Utrecht, The Netherlands

[9] Health Effects Institute, Cambridge, MA, USA

[10] The Chinese University of Hong Kong, Hong Kong

[11] Goddard Earth Sciences Technology and Research, Universities Space Research Association, Columbia, MD, USA

[12] NASA Goddard Space Flight Center, Greenbelt, MD, USA.

[13] Harvard-Smithsonian Center for Astrophysics, Cambridge, MA

Table of Contents

ISAAC Phase One and Phase Three Study Groups....……………………………………...3

Supplemental Material, Figure S1: Scatter plots of the association of asthma prevalence ages 6-7 years with a) $PM_{2.5}$ (µg/m^3) and b) NO_2 (ppbV)…………..………....7

Supplemental Material, Figure S2: Flow diagram illustrating sample attrition…………8

Supplemental Material, Table S1: Descriptive statistics for the main analytic dataset ages 6-7 years……………………………………………………....…...……...9

Supplemental Material, Table S2: The association of asthma prevalence ages 6-7 years with $PM_{2.5}$ and NO_2……………………………….……….….…....10

References……………………........……………………………………….………...11

ISAAC Phase One and Phase Three Study Group

ISAAC Steering Committee:

N Aït-Khaled* (International Union Against Tuberculosis and Lung Diseases, Paris, France); HR Anderson (Division of Population Health Sciences and Education, St Georges, University of London, London, UK); MI Asher (Department of Paediatrics: Child and Youth Health, Faculty of Medical and Health Sciences, The University of Auckland, New Zealand); R Beasley* (Medical Research Institute of New Zealand, Wellington, New Zealand); B Björkstén* (Institute of Environmental Medicine, Karolinska Institutet, Stockholm, Sweden); B Brunekreef (Institute of Risk Assessment Science, Universiteit Utrecht, Netherlands); J Crane (Wellington Asthma Research Group, Wellington School of Medicine, New Zealand); P Ellwood (Department of Paediatrics: Child and Youth Health, Faculty of Medical and Health Sciences, The University of Auckland, New Zealand); C Flohr (Department of Paediatric Allergy & Dermatology, St John's Institute of Dermatology, London, UK); S Foliaki* (Centre for Public Health Research, Massey University, Wellington, New Zealand); F Forastiere (Department of Epidemiology, Rome E Health Authority, Rome, Italy); L García-Marcos (Respiratory Medicine and Allergy Units, 'Virgen de la Arrixaca' University Children's Hospital, University of Murcia, Spain); U Keil* (Institut für Epidemiologie und Sozialmedizin, Universität Münster, Germany); CKW Lai* (Department of Medicine and Therapeutics, The Chinese University of Hong Kong SAR, China); J Mallol* (Department of Paediatric Respiratory Medicine, University of Santiago de Chile, Chile); EA Mitchell (Department of Paediatrics: Child and Youth Health, Faculty of Medical and Health Sciences, The University of Auckland, New Zealand); S Montefort* (Department of Medicine, University of Malta, Malta), J Odhiambo* (Centre Respiratory Diseases Research Unit, Kenya Medical Research Institute, Nairobi, Kenya); N Pearce (Department of Medical Statistics, Faculty of Epidemiology and Public Health, London School of Hygiene and Tropical Medicine, London, UK); CF Robertson (Murdoch Children's Research Institute, Melbourne, Australia); AW Stewart (Population Health, Faculty of Medical and Health Sciences, The University of Auckland, New Zealand); D Strachan (Division of Population Health Sciences and Education, St Georges, University of London, London, UK); E von Mutius (Dr von Haunerschen Kinderklinik de Universität München, Germany); SK Weiland[†] (Department of Epidemiology, University of Ulm, Germany); G Weinmayr (Institute of Epidemiology, University of Ulm, Germany); H Williams (Centre for Evidence Based Dermatology, Queen's Medical Centre, University Hospital, Nottingham, UK); G Wong (Department of Paediatrics, Prince of Wales Hospital, Hong Kong SAR, China).
* Regional Coordinator; [†] Deceased.

ISAAC International Data Centre:

MI Asher, TO Clayton, E Ellwood, P Ellwood, EA Mitchell, Department of Paediatrics: Child and Youth Health, and AW Stewart, School of Population Health, Faculty of Medical and Health Sciences, The University of Auckland, New Zealand.

ISAAC Phase One Principal Investigators:
Albania: Prof A Priftanji* (Tiranë); Australia: Prof CF Robertson* (Melbourne); Austria: Assoc Prof G Haidinger* (Urfahr-Umgebung); Belgium: Prof P Vermeire (Antwerp); Brazil: Dr PGM Bezerra (Recife), Prof N Rosário (Curitiba), Prof D Solé* (São Paulo), Prof R Stein (Porto Alegre), Assoc Prof L de Freitas Souza (Salvador); Canada: Dr B Taylor (Saskatoon); Channel Islands: Dr R Grainger (Jersey), Dr D Jeffs (Guernsey); Chile: Dr L Amarales (Punta Arenas), Dr MA Calvo (Valdivia), Dra E Cortez (South Santiago); China: Prof Y-Z Chen* (Beijing), Prof N-S Zhong (Guangzhou); Estonia: Dr M-A Riikjärv* (Tallinn); Ethiopia: Assoc Prof K Melaku (Addis Ababa); Georgia: Dr N Khetsuriani (Kutaisi); Germany: Prof Dr U Keil* (Münster); Hong Kong: Dr CKW Lai* (Hong Kong), Prof YL Lau (Hong Kong); India: Dr K Chopra (New Delhi (7)), Dr NM

Hanumante (Pune), Dr KC Jain (Jodhpur), Dr MK Joshi (Bombay (16)), Dr VA Khatav (Borivali), Prof L Kumar (Chandigarh), Dr K Raghavan (Bombay (18)), Dr N Somu (Madras (3)), Dr TU Sukumaran (Kottayam); Indonesia: Prof Dr K Baratawidjaja* (Bandung); Iran: Dr M-R Masjedi* (Rasht, Tehran); Isle of Man: Dr P Powell (Isle of Man); Italy: Dr L Bisanti (Milano), Dr E Bonci (Cosenza), Dr E Chellini (Firenze), Ms L Chetoni (Empoli), Dr G Ciccone (Torino), Dr F Forastiere* (Roma); Japan: Prof S Nishima* (Fukuoka); Kenya: Dr FO Esamai (Eldoret), Dr JA Odhiambo (Nairobi); Kuwait: Dr JA al-Momen (Kuwait); Latvia: Dr M Leja (Riga); Malaysia: Prof BS Quah (Kota Bharu), Dr KH Teh (Alor Setar); Malta: Prof S Montefort* (Malta); Mexico: Prof I Romieu (Cuernavaca); Morocco: Prof Z Bouayad* (Casablanca, Marrakech); New Zealand: Prof J Crane (Wellington), Dr R MacKay (Nelson), Assoc Prof P Pattemore (Christchurch); Nigeria: Prof BO Onadeko (Ibadan); Pakistan: Dr ZA Bhutta (Karachi); Paraguay: Dr JA Guggiari-Chase* (Asunción); Peru: Dr P Chiarella* (Lima); Philippines: Prof F Cua-Lim* (Metro Manila); Poland: Assoc Prof A Brêborowicz (Poznan), Assoc Prof G Lis* (Kraków); Portugal: Dr FD Borges (Funchal), Dr JM Lopes dos Santos (Porto), Dr C Nunes (Portimao); Romania: Prof D Deleanu* (Cluj); Singapore: Prof B-W Lee* (Singapore); South Korea: Dr S-I Lee (Seoul); Spain: Dr A Arnedo-Pena (Castellón), Dr RM Busquets (Barcelona), Prof L García-Marcos* (Cartagena), Prof F Guillén-Grima (Pamplona), Prof MM Morales Suárez-Varela (Valencia), Dr AD Rubio (Bilbao); Sweden: Prof N-IM Kjellman (Linköping); Taiwan: Prof K-H Hsieh (Taipei); Thailand: Assoc Prof M Trakultivakorn (Chiang Mai); USA: Prof GJ Redding (Seattle); United Kingdom: Dr MH Shamssain (Sunderland); Uruguay: Dra D Holgado* (Montevideo).
* National Coordinator.

ISAAC Phase Three Principal Investigators:
Albania: Prof A Priftanji* (Tiranë); Algeria: Prof B Benhabylès (Wilaya of Algiers); Argentina: Dr CE Baena-Cagnani* (Córdoba), Prof Dr CD Crisci (Rosario City), Dr M Gómez (Salta), Prof GE Zabert (Neuquén); Australia: Prof CF Robertson* (Melbourne); Austria: Assoc Prof G Haidinger* (Kärnten, Urfahr-Umgebung); Barbados: Dr ME Howitt* (Barbados); Belgium: Prof J Weyler (Antwerp); Bolivia: Dr R Pinto-Vargas* (Santa Cruz); Brazil: Dr CdSD Bernhardt (Itajaí), Dr WG Borges (Brasília), Prof PAM Camargos (Belo Horizonte), Dra MdS Cardoso (Manaus Amazonas), Prof AJLAd Cunha (Nova Iguaçu), Dr GB Fischer (Porto Alegre), Dr JM Motta (Aracaju), Prof FJ Passos (Maceió), Dr AC Pastorino (São Paulo West), Dr AC Porto Neto (Passo Fundo), Prof N Rosário (Curitiba), Assis Prof A Silva (Caruaru), Prof D Solé* (Rural Santa Maria, Santa Maria, São Paulo), Assoc Prof N Wandalsen (Santo Andre), Dr M de Britto (Recife), Assoc Prof L de Freitas Souza (Feira de Santana, Salvador, Vitória da Conquista); Bulgaria: Dr T Popov* (Sofia); Cameroon: Prof C Kuaban* (Yaounde); Canada: Prof A Ferguson (Vancouver), Prof D Rennie (Saskatoon); Channel Islands: Ms R Goulding (Jersey), Dr P Standring (Guernsey); Chile: Dr P Aguilar (South Santiago), Dr L Amarales (Punta Arenas), Dr LAV Benavides (Calama), Dr MA Calvo (Valdivia), Dra A Contreras (Chiloe); China: Prof Y-Z Chen* (Beijing, Tong Zhou), Assis Prof O Kunii (Tibet), Dr Q Li Pan (Wulumuqi), Prof N-S Zhong (Guangzhou); Colombia: Dr G Aristizábal (Bogotá), Dr AM Cepeda (Barranquilla), Dr GA Ordoñez (Cali); Congo: Prof J M'Boussa (Brazzaville); Cook Islands: Dr R Daniel* (Rarotonga); Cote d'Ivoire: Dr BN Koffi* (Urban Cote d'Ivoire); Croatia: Dr K Lah Tomulic (Rijeka); Cuba: Dra P Varona Peréz* (La Habana); Ecuador: Dr S Barba* (Quito), Dr C Bustos (Guayaquil); Egypt: Dr ML Naguib (Cairo); El Salvador: Dr M Figueroa Colorado* (San Salvador); Estonia: Dr M-A Riikjärv* (Tallinn); Ethiopia: Assoc Prof K Melaku (Addis Ababa); Fiji: Dr R Sa'aga-Banuve (Suva); Former Yugoslav Republic of Macedonia (FYROM): Assoc Prof E Vlaski* (Skopje); Gabon: Dr IE Hypolite* (Port-Gentil); Georgia: Dr M Gotua* (Kutaisi); Germany: Prof Dr U Keil* (Münster); Greece: Assoc Prof J Tsanakas (Thessaloniki); Honduras: Dr A Bueso-Engelhardt* (San Pedro Sula); Hong Kong: Prof YL Lau (Hong Kong), Prof G Wong (Hong Kong); Hungary: Dr Z Novák (Szeged), Dr G Zsigmond* (Svábhegy); India: Prof S Awasthi (Lucknow), Assoc Prof S Bhave (Rasta Peth), Prof J Chhatwal (Ludhiana), Dr NM Hanumante (Pune), Dr KC Jain (Jodhpur), Dr MK Joshi (Mumbai (16)), Dr VA Khatav (Borivali), Prof L Kumar (Chandigarh), Dr SN Mantri (Mumbai (29)), Dr AV

Pherwani (Mumbai (18)), Prof S Rego (Bangalore), Prof M Sabir (Bikaner), Dr S Salvi (Nagpur, Pimpri), Dr G Setty (Chennai (3)), Prof SK Sharma (New Delhi (7)), Prof V Singh (Jaipur), Dr TU Sukumaran (Kottayam), Dr PS Suresh Babu (Davangere); Indonesia: Prof Dr CB Kartasasmita (Bandung), Prof P Konthen (Bali), Dr W Suprihati (Semarang); Iran: Dr M-R Masjedi* (Birjand, Rasht, Tehran, Zanjan); Isle of Man: Dr A Steriu (Isle of Man); Italy: Dr L Armenio (Bari), Dr L Bisanti (Milano), Dr E Bonci (Cosenza), Dr E Chellini (Firenze), Dr G Ciccone (Torino), Dr V Dell'Orco (Colleferro-Tivoli), Dr F Forastiere* (Roma), Dr G Giannella (Mantova), Dr S La Grutta (Palermo), Dr MG Petronio (Empoli); Japan: Dr H Odajima (Fukuoka), Prof M Sohei (Tochigi); Jordan: Dr F Abu-Ekteish (Amman); Kenya: Dr FO Esamai (Eldoret), Dr L Ng'ang'a* (Nairobi); Kingdom of Tonga: Dr S Foliaki (Nuku'alofa); Kuwait: Dr JA al-Momen (Kuwait); Kyrgyzstan: Dr C Imanalieva* (Balykchi, Bishkek), Prof S Sulaimanov (Jalalabat); Latvia: Dr V Svabe (Riga); Lithuania: Prof J Bojarskas (Panevezys, Siauliai), Assoc Prof J Kudzyte* (Kaunas); Malaysia: Prof BS Quah (Kota Bharu), Dr KH Teh (Alor Setar); Malta: Prof S Montefort* (Malta); Mexico: Dr M Baeza-Bacab* (Mérida), Dra M Barragán-Meijueiro (Ciudad de México (3)), Dra BE Del-Río-Navarro (Ciudad de México (1)), Dr R García-Almaráz (Ciudad Victoria), Dr SN González-Díaz (Monterrey), Dr FJ Linares-Zapién (Toluca), Dr JV Merida-Palacio (Mexicali Valley), Dra N Ramírez-Chanona (Ciudad de México (4)), Dr S Romero-Tapia (Villahermosa), Prof I Romieu (Cuernavaca); Morocco: Prof Z Bouayad* (Benslimane, Boulmene, Casablanca, Marrakech); Netherlands: Prof R Engels (Netherlands); New Zealand: Dr R MacKay (Nelson), Assoc Prof P Pattemore (Christchurch), Prof N Pearce (Wellington); Nicaragua: Dr JF Sánchez* (Managua); Nigeria: Prof BO Onadeko (Ibadan); Niue: Ms M Magatogia (Niue Island); Nouvelle Caledonie: Dr I Annesi-Maesano (Nouvelle Caledonie); Pakistan: Dr N Mahmood* (Karachi), Dr MO Yusuf (Islamabad); Palestine: Dr N El Sharif* (Ramallah), Mr S Mortaja (North Gaza); Paraguay: Dr JA Guggiari-Chase* (Asunción); Peru: Dr P Chiarella* (Lima); Philippines: Prof F Cua-Lim* (Metro Manila); Poland: Assoc Prof A Brêborowicz (Poznan), Assoc Prof G Lis* (Kraków); Polynesie Francaise: Dr I Annesi-Maesano (Polynesie Francaise); Portugal: Dr ML Chiera (Coimbra), Dra R Câmara (Funchal), Dr JM Lopes dos Santos (Porto), Dr C Nunes (Portimao); Republique Democratique du Congo: Prof Dr J-M Kayembe (Kinshasa); Reunion Island: Dr I Annesi-Maesano (Reunion Island); Romania: Prof D Deleanu* (Cluj); Russia: Prof Dr EG Kondiourina (Novosibirsk); République de Guinée: Prof OY Sow (Conakry); Samoa: Ms P Fuimaono V Pisi (Apia); Serbia and Montenegro: Dr O Adzovic (Podgorica), Dr M Hadnadjev (Novi Sad), Dr E Panic (Sombor), Dr S Zivanovic (Nis), Dr Z Zivkovic* (Belgrade); Singapore: Assoc Prof DYT Goh (Singapore); South Africa: Prof K Voyi (Polokwane); South Korea: Prof H-B Lee* (Seoul); Spain: Dr A Arnedo-Pena (Castellón), Dr J Batlles-Garrido (Almeria), Dr RM Busquets (Barcelona), Dr I Carvajal-Urueña (Asturias), Dr G García-Hernández (Madrid), Prof L García-Marcos* (Cartagena), Dr C González Díaz (Bilbao), Prof F Guillén-Grima (Pamplona), Dr A López-Silvarrey Varela (A Coruña), Prof MM Morales Suárez-Varela (Valencia), Prof EG Pérez-Yarza (San Sebastián); Sri Lanka: Dr KD Gunasekera* (Sri Lanka); Sudan: Prof OAA Musa (Khartoum); Sultanate of Oman: Assoc Prof O Al-Rawas* (Al-Khod); Sweden: Dr H Vogt (Linköping); Syrian Arab Republic: Dr S Mohammad* (Tartous), Prof Y Mohammad (Lattakia), Dr K Tabbah (Aleppo); Taiwan: Dr J-L Huang* (Taipei), Dr C-C(Kao (Taoyuan); Thailand: Dr A Kongpanichkul (Nakorn Pathom), Dr R Nettagul (Chiangrai), Dr T Prasarnphanich (Chantaburi), Assoc Prof J Teeratakulpisarn (Khon Kaen), Assoc Prof M Trakultivakorn (Chiang Mai); Togo: Prof O Tidjani (Lome); Tokelau: Dr T Iosefa* (Tokelau); Trinidad and Tobago: Dr MA Monteil (St Augustine, Tobago); Tunisia: Prof M Jerray (Sousse), Prof F Khaldi (Grand Tunis); USA: Prof GJ Redding (Seattle), Dr HH Windom (Sarasota), Dr K Yeatts (Chapel Hill); Ukraine: Assoc Prof V Ognev* (Kharkiv, Rural Kharkiv); United Kingdom: Dr MH Shamssain (Sunderland); Uruguay: Dra D Holgado* (Montevideo), Dra MC Lapides (Paysandú); Venezuela: Dr O Aldrey* (Caracas); Vietnam: Dr B Vaên Cam (Ho Chi Minh City).

* National Coordinator.

ISAAC National Coordinators not identified above:

Canada: M Sears; Channel Islands: HR Anderson; Chile: V Aguirre, J Mallol; Colombia: J Mallol (interim); Costa Rica: ME Soto-Quirós; Croatia: V Ahel; Fiji: L Waqatakirewa; Finland: J Pekkanen; Greece: C Gratziou; India: J Shah; Isle of Man: HR Anderson; Kingdom of Tonga: T Fakakovi; Malaysia: J de Bruyne; Netherlands: R Otten; New Zealand: MI Asher; Nouvelle Caledonie: S Barny; Panama: G Cukier; Polynesie Francaise: R Chansin; Portugal: J Rosado Pinto; Republic of Ireland: P Manning; Republique Democratique du Congo: E Bahati; Reunion Island: C Catteau; Russia: RM Khaitov; Samoa: N Tuuau-Potai; Sudan: A El Sony; Sweden: L Nilsson; South Africa: HJ Zar; Thailand: P Vichyanond; United Kingdom: HR Anderson.

a)

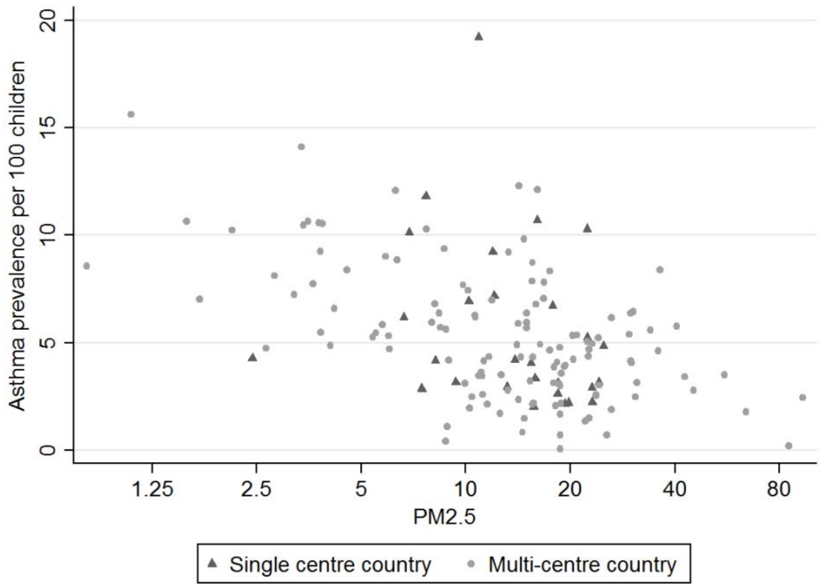

b)

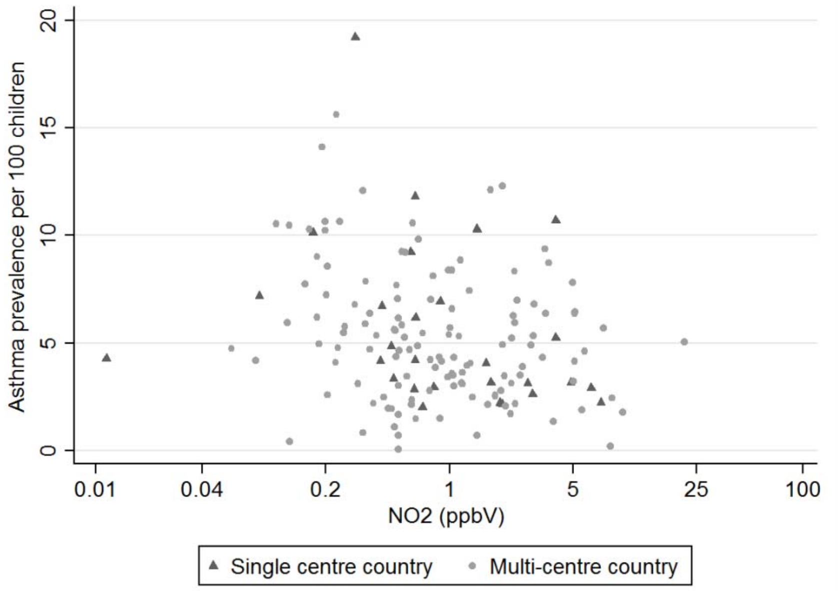

Supplemental Material, Figure S1:
Scatter plots of the association of asthma prevalence ages 6-7 years with a) $PM_{2.5}$ µg/m^3 and b) NO_2 (ppbV)

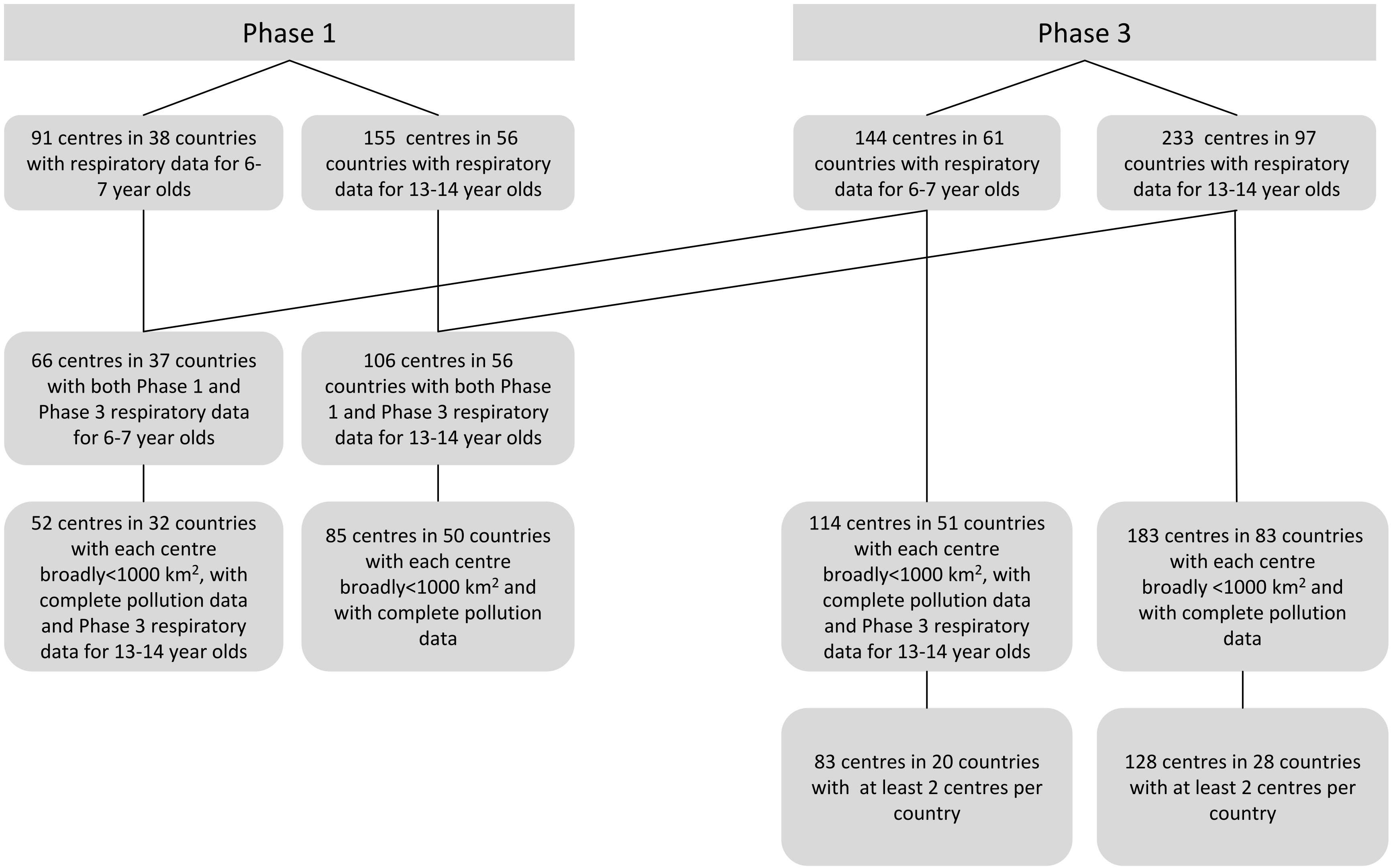

Supplemental Material, Figure S2:
Flow diagram illustrating sample attrition (data from Lai et al.2009, Pearce et al.2007)

Supplemental Material, Table S1: Descriptive statistics for the main analytic dataset ages 6-7 years

Variables	Time period	Median(IQ range)	Spearman correlation with: Asthma prevalence	$PM_{2.5}$	NO_2	O_3
Full dataset: 114 centres clustered within 51 countries						
Sex (ISAAC Phase Three, ages 6-7 years)						
% boys in sample	≈2000-2003	50.4 (48.7 – 52.0)	-0.221*	0.256**	0.094	0.266**
Disease (ISAAC Phase Three, ages 6-7 years)						
Asthma prevalence (%)	≈2000-2003	3.86 (2.53 – 6.11)		-0.445***	-0.204*	-0.475***
Climate / Altitude						
Daily temperature (˚c)	1991-2000	17.6 (12.4 – 24.9)	0.116	0.031	-0.321***	-0.001
Water vapour pressure (hPa)	1991-2000	14.0 (10.4 – 22.1)	0.186*	-0.114	-0.323***	-0.143
Precipitation (mm/month)	1991-2000	75.9 (50.3 – 121.1)	0.148	-0.242**	-0.175	-0.321***
Altitude (m)	N/A	76.5 (20 – 450)	-0.094	0.223*	0.061	0.131
Economic / population						
GNI per capita (US $)	2001	3410 (1170 – 12511)	-0.067[a]	-0.057[a]	0.490[a]***	-0.070[a]
Population density (thousands per 0.1˚ x 0.1˚ grid square)	2005	162 (53.0 – 544)	-0.037	0.278**	0.398***	0.207*
Pollution						
$PM_{2.5}$ (µg/m^3)	2001-2006	15.5 (10.2 – 20.4)			0.405***	0.565***
NO_2 (ppbV)	2005	0.89 (0.47 – 2.22)				0.371***
O_3 (ppbV)	2005	53.5 (41.2 – 62.4)				

* p<0.05, ** p<0.01, *** p<0.001.

[a] Correlations for GNI per capita (available only at country-level) are with country-level variables (defined as in manuscript formula 1.1). There are 51 countries. All other correlations are across all 114 centres.

Supplemental Material, Table S2: The association of asthma prevalence ages 6-7 years with $PM_{2.5}$ and NO_2.

			Estimated change in centre-level asthma prevalence (95% CI) per 100 children per 10% increase in:			
			$PM_{2.5}$		NO_2	
No.	Model type:	Adjustment	Country-level[a]	Centre-level[b]	Country-level[a]	Centre-level[b]
**Using data from 114 centres in 51 countries**						
1	Random intercept	Unadjusted	-0.169 (-0.352 to 0.014)	-0.041 (-0.167 to 0.085)	-0.048 (-0.139 to 0.043)	-0.015 (-0.065 to 0.035)
2	Random intercept	Sex, climate, GNI	-0.256* (-0.452 to -0.060)	-0.016 (-0.152 to 0.121)	-0.040 (-0.149 to 0.068)	-0.019 (-0.071 to 0.033)
3	Random intercept	Sex, climate, GNI, population density	-0.306** (-0.514 to -0.098)	0.026 (-0.120 to 0.172)	-0.059 (-0.188 to 0.071)	0.004 (-0.059 to 0.068)
**Restricted to two or more centres per country (83 centres in 20 countries)**						
4	Random intercept	Sex, climate, GNI, population density	-0.430*** (-0.668 to -0.191)	0.026 (-0.116 to 0.168)	0.046 (-0.147 to 0.240)	0.004 (-0.059 to 0.067)
5	Random intercept/random slope[c]	Sex, climate, GNI, population density	-0.430*** (-0.669 to -0.191)	0.026 (-0.117 to 0.168)	0.049 (-0.144 to 0.243)	-0.009 (-0.073 to 0.055)
6	Random intercept	Sex, climate, GNI, population density, O_3	-0.356 (-0.767 to 0.055)	0.030 (-0.108 to 0.167)	0.130 (-0.027 to 0.287)	0.017 (-0.044 to 0.079)
7	Random intercept	Sex, climate, GNI, population density, log($PM_{2.5}$) or log(NO_2) as appropriate	-0.475*** (-0.717 to -0.232)	0.028 (-0.136 to 0.192)	0.125 (-0.023 to 0.274)	-0.002 (-0.074 to 0.071)
8	Random intercept	Sex, climate, GNI, population density, O_3, log($PM_{2.5}$) or log(NO_2) as appropriate	-0.320 (-0.705 to 0.066)	0.014 (-0.144 to 0.172)	0.124 (-0.027 to 0.275)	0.014 (-0.057 to 0.086)

*p<0.05; **p<0.01; ***p<0.001

[a]Country-level effect: estimate of the association between centre-level asthma prevalence and country-level pollutant (defined as in manuscript formula 1.1).

[b]Centre-level effect: estimate of the within country association between centre-level asthma prevalence and centre-level pollutant.

[c] Test for a random slope in $PM_{2.5}$ (Model 5), χ^2=0.005 (degrees of freedom=2) p>0.05; test for a random slope in NO_2 (Model 5), χ^2=2.09 (degrees of freedom=2) p>0.05.

Note: With the exception of GNI per capita (available only at country-level), sex, climate (i.e. temperature, precipitation, water vapour pressure), population density and pollutants, if included in models were included both as country-level (defined as in manuscript formula 1.1) and centre-level variables.

References

Lai CK, Beasley R, Crane J, Foliaki S, Shah J, Weiland S. 2009. Global variation in the prevalence and severity of asthma symptoms: phase three of the International Study of Asthma and Allergies in Childhood (ISAAC). Thorax 64:476-83.

Pearce N, Ait-Khaled N, Beasley R, Mallol J, Keil U, Mitchell E, et al. 2007. Worldwide trends in the prevalence of asthma symptoms: phase III of the International Study of Asthma and Allergies in Childhood (ISAAC). Thorax 62:758-66.
